# Nuclear Envelope Alterations in Myotonic Dystrophy Type 1 Patient-Derived Fibroblasts

**DOI:** 10.3390/ijms23010522

**Published:** 2022-01-04

**Authors:** Diana Viegas, Cátia D. Pereira, Filipa Martins, Tiago Mateus, Odete A. B. da Cruz e Silva, Maria Teresa Herdeiro, Sandra Rebelo

**Affiliations:** Department of Medical Sciences, Institute of Biomedicine (iBiMED), University of Aveiro, 3810-193 Aveiro, Portugal; dianaviegas97@ua.pt (D.V.); daniela.pereira@ua.pt (C.D.P.); samartins@ua.pt (F.M.); tiago12@ua.pt (T.M.); odetecs@ua.pt (O.A.B.d.C.e.S.); teresaherdeiro@ua.pt (M.T.H.)

**Keywords:** myotonic dystrophy type 1, nuclear envelope, DMPK, nuclear profile, lamin A/C, emerin, LAP1, SUN1, nesprin-1, nesprin-2

## Abstract

Myotonic dystrophy type 1 (DM1) is a hereditary and multisystemic disease characterized by myotonia, progressive distal muscle weakness and atrophy. The molecular mechanisms underlying this disease are still poorly characterized, although there are some hypotheses that envisage to explain the multisystemic features observed in DM1. An emergent hypothesis is that nuclear envelope (NE) dysfunction may contribute to muscular dystrophies, particularly to DM1. Therefore, the main objective of the present study was to evaluate the nuclear profile of DM1 patient-derived and control fibroblasts and to determine the protein levels and subcellular distribution of relevant NE proteins in these cell lines. Our results demonstrated that DM1 patient-derived fibroblasts exhibited altered intracellular protein levels of lamin A/C, LAP1, SUN1, nesprin-1 and nesprin-2 when compared with the control fibroblasts. In addition, the results showed an altered location of these NE proteins accompanied by the presence of nuclear deformations (blebs, lobes and/or invaginations) and an increased number of nuclear inclusions. Regarding the nuclear profile, DM1 patient-derived fibroblasts had a larger nuclear area and a higher number of deformed nuclei and micronuclei than control-derived fibroblasts. These results reinforce the evidence that NE dysfunction is a highly relevant pathological characteristic observed in DM1.

## 1. Introduction

Myotonic dystrophy type 1 (DM1) is the most common adult-onset muscular dystrophy with an estimated prevalence of 1:8000 [1,2]. DM1 is characterized by a slowly progressing muscle weakness, loss of muscle mass and myotonia. Additionally, DM1 is also categorized as a multisystemic disease, affecting other organs, namely the eyes (cataracts), heart (conduction problems leading to cardiomyopathies) and respiratory system, and causing metabolic alterations (insulin insensitivity and diabetes) [3,4,5,6]. DM1 is a genetic disease caused by an abnormal unstable expansion of the CTG trinucleotide in the 3′UTR of the *Myotonic Dystrophy Protein Kinase (DMPK)* gene [1,7]. The central protein of DM1, DMPK, is a protein kinase that consists of seven distinct isoforms (DMPK A to G) in humans, which are generated by alternative splicing. DMPK’s subcellular localization is confined to either the endoplasmic reticulum or nuclear envelope (NE) (DMPK A and B), mitochondria (DMPK C and D) or cytoplasm (DMPK E, F and G) [6,8,9].

Several studies have been carried out to unravel the molecular mechanisms underlying this pathology. To date, there are three more consensual hypotheses explaining the pathogenesis of DM1: RNA toxic gain-of-function, haploinsufficiency of DMPK and rearrangement of the DM1 locus [6,10,11,12]. Despite the great deal of effort for unravelling the molecular mechanism underlying DM1, none of these hypotheses can explain all the multisystemic signs and symptoms. A defect in the positioning of myonuclei, resulting from alterations in nuclear envelope (NE) proteins, has also been proposed as a potential pathological mechanism of DM1, similar to other muscular dystrophies [13,14,15,16]. Previous studies have reported that some muscular dystrophies result from alterations in NE stability [13,14,15,16]. A common feature of these diseases is the presence of nuclei usually located and grouped in the muscle cells’ center, compromising myonuclear movement [13,17]. NE proteins are essential for gene regulation, nuclear structure and muscle function [18,19]. In the case of DM1, very few studies have been carried out to assess alterations of NE proteins in DM1 [20,21,22], and the contribution of NE dysfunction to DM1 has not been fully elucidated.

Therefore, the main objectives of this study were to evaluate the nuclear profile in DM1 patient-derived and control fibroblasts and to determine the intracellular protein levels and immunolocalization of the disease-associated DMPK protein and other NE proteins, namely lamin A/C, emerin, lamin-associated polypeptide 1 (LAP1), Sad1/unc-84 protein-like (SUN1), nesprin-1 and nesprin-2, in both cell lines. The results obtained here may provide new insights on the potential contribution of NE dysfunction to DM1 pathogenesis. 

## 2. Results

### 2.1. Evaluation of Intracellular DMPK Protein Levels in DM1 Patient-Derived Fibroblasts

The precise molecular mechanism underlying DM1 is still elusive. The toxic gain of function of expanded CUG repeats of mutant *DMPK* mRNA and haploinsufficiency are two well accepted proposed mechanisms [6]. As a consequence, the protein levels of DMPK are found to be decreased in DM1 tissues [23]. To confirm these changes, intracellular DMPK protein levels were evaluated by immunoblotting in DM1 patient-derived and control fibroblasts. Briefly, in this study, two cell lines were used with approximately 1000 CTG repeats, hereafter referred to as DM1_1000 (1) and DM1_1000 (2), two cell lines with approximately 2000 CTG repeats, hereafter designated DM1_2000 (1) and DM1_2000 (2), and one control cell line that comprised between 5 and 27 CTG repeats.

**Figure 1:**
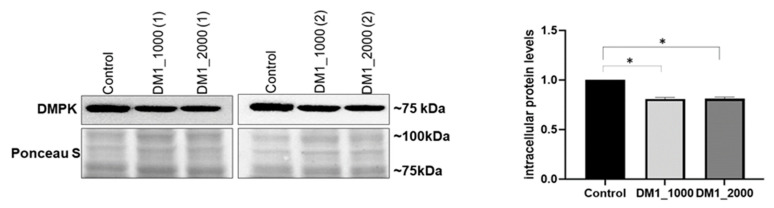
Intracellular DMPK protein levels in DM1 patient-derived and control fibroblasts. The intracellular protein levels in DM1 patient-derived fibroblasts were estimated in relation to the protein levels detected in the control condition and are presented as mean ± SEM of four independent experiments. Ponceau S staining was used to assess gel loading. The statistical analysis was performed using one-way ANOVA followed by the Tukey’s multiple comparison test, used to compare between DM1_1000, DM1_2000 and the control groups. * *p* < 0.05; DM1—myotonic dystrophy type 1; DMPK—myotonic dystrophy protein kinase; SEM—standard error of the mean.

The results presented in Figure 1 show that the intracellular DMPK protein levels were significantly decreased in the DM1_1000 (*p* = 0.0332) and DM1_2000 (*p* = 0.0332) fibroblasts when compared to the control (Figure 1).

As expected, significantly lower levels of DMPK protein were observed in the DM1 patient-derived fibroblasts. Therefore, we decide to further explore the contribution of NE dysfunction to DM1 through the evaluation of the nuclear profile as well as protein levels and the subcellular distribution of several relevant NE proteins.

### 2.2. Evaluation of the Nuclear Profile in DM1 Patient-Derived Fibroblasts

**Figure 2:**
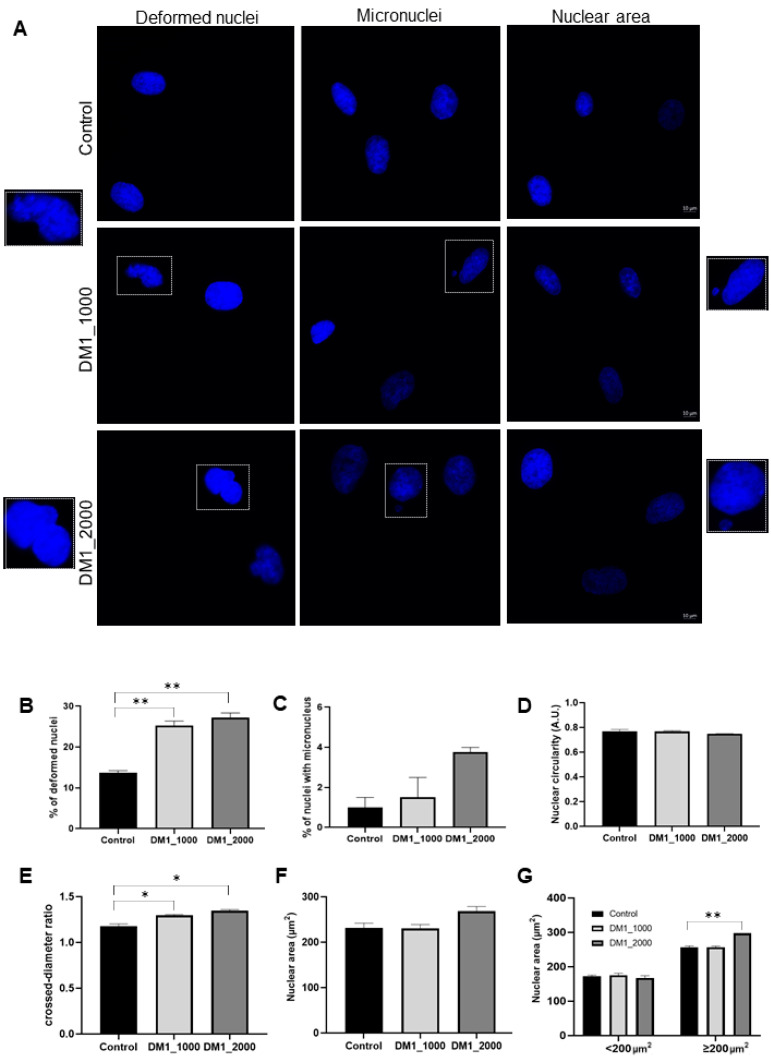
Nuclear profile of DM1 patient-derived and control fibroblasts. (**A**) The nuclear profiles of DM1 patient-derived and control fibroblasts were analysed using fluorescence microscopy and representative images are presented. Fibroblasts’ nuclei were stained with DAPI (blue). Quantitative evaluation of (**B**) deformed nuclei, (**C**) micronuclei, (**D**) nuclear circularity, (**E**) crossed diameter ratio, (**F**) nuclear area comparison between DM1 patient-derived fibroblasts (DM1_1000 and DM1_2000) and control group and (**G**) nuclear area of <200 µm^2^ and ≥200 µm^2^. The quantitative data are presented as mean ± SEM and were obtained by analysing 100 cells per condition from four independent experiments. The statistical analysis was performed using one-way ANOVA followed by Tukey’s multiple comparison test used to compare between DM1_1000, DM1_2000 and the control groups. * *p* < 0.05, ** *p* < 0.01. Scale bar = 10 µm. A.U.—arbitrary units; DM1—myotonic dystrophy type 1; SEM—standard error of the mean.

The presence of nuclear architectural alterations in DM1 patient-derived fibroblasts was assessed through DAPI staining, followed by the monitoring of several nuclear parameters, namely the occurrence of nuclear deformations, number of micronuclei, nuclear circularity, crossed diameter ratio and nuclear area (Figure 2). Nuclear circularity is a quantitative measure that assesses the circular shape of nuclei, with a maximum value of 1 corresponding to a perfect circle. Concerning nuclear deformations, the existence of blebs, lobed nuclei, micronuclei and nuclear invaginations were taken into consideration. Several visible small nuclei were also quantified as micronuclei.

Regarding the presence of nuclear deformations (Figure 2A,B), there was a significant increase in the percentage of deformed nuclei in DM1 patient-derived fibroblasts (DM1_1000, *p* = 0.0066; DM1_2000, *p* = 0.0012) relative to control fibroblasts. The results indicated that DM1 patient-derived fibroblasts carrying a higher number of CTG repeats seemed to present a higher number of nuclear deformations (Figure 2B). The number of micronuclei also seemed to increase in DM1 patient-derived fibroblasts in comparison to control fibroblasts (Figure 2C) and was correlated with CTG repeat length. Regarding the crossed diameter ratio, this parameter was significantly increased in the fibroblast nuclei derived from DM1_1000 (*p* = 0.0328) and DM1_2000 (*p* = 0.0127) fibroblasts when compared with the control fibroblasts (Figure 2E). Finally, the mean nuclear area of DM1 patient-derived fibroblasts appeared to be larger than the control (Figure 2F). Knowing that the average nuclear area of fibroblasts is around 200 µm^2^ [24] (Figure 2G), we quantified the number of cells with a nuclear area <200 µm^2^ and ≥ 200 µm^2^. Interestingly, it was found that there was a significant increase in the nuclear area in DM1_2000 fibroblasts compared to the control (*p* = 0.0041) (Figure 2G).

Since important nuclear changes were observed in DM1 patient-derived fibroblasts in relation to control fibroblasts, it seemed important to evaluate some relevant proteins of the NE. 

### 2.3. Evaluation of Intracellular Levels and Localization of NE Proteins in DM1 Patient-Derived Fibroblasts

**Figure 3:**
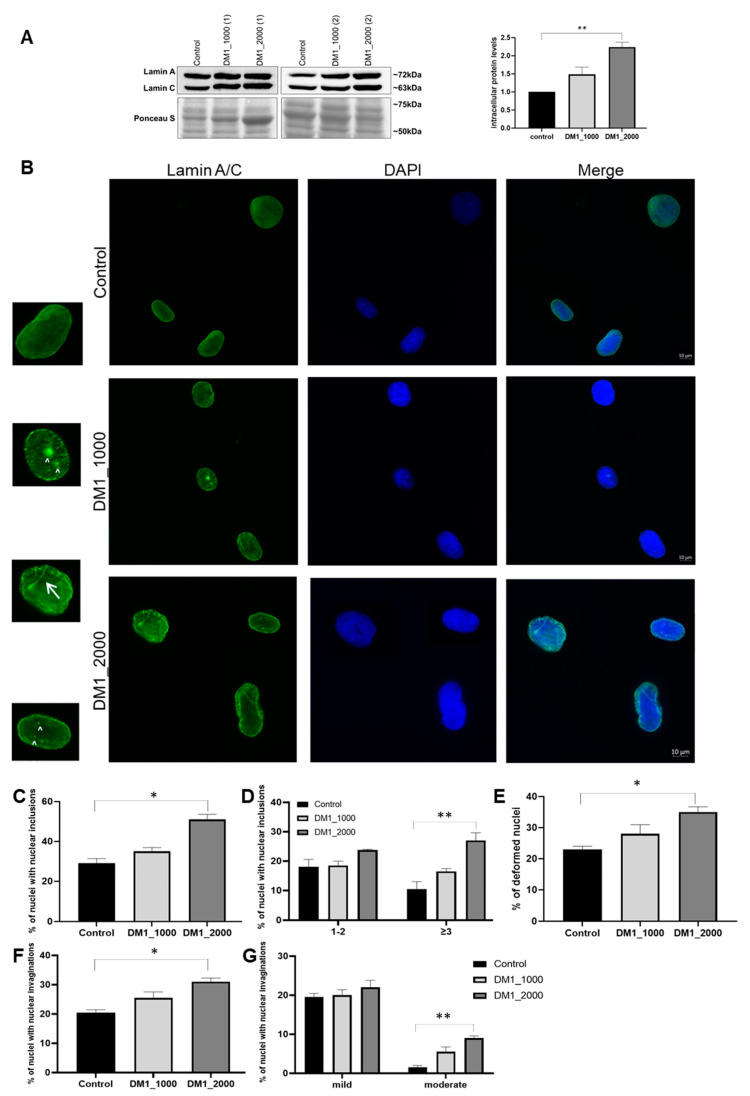
Intracellular protein levels and nuclear localization of lamin A/C in DM1 patient-derived and control fibroblasts. (**A**) Intracellular lamin A/C protein levels in DM1 patient-derived and control fibroblasts were analysed using immunoblotting. The intracellular protein levels in DM1 patient-derived fibroblasts were estimated in relation to protein levels detected in the control condition and are presented as mean ± SEM of four independent experiments. Ponceau S staining was used to assess gel loading. The statistical analysis was performed using one-way ANOVA followed by Tukey’s multiple comparison test to compare between DM1_1000, DM1_2000 and the control groups. To compare intracellular protein levels between groups, one-way ANOVA was used, followed by Tukey’s multiple comparison test. ** *p* < 0.01 (**B**) Subcellular distribution of lamin A/C in DM1 patient-derived and control fibroblasts was analysed using fluorescence microscopy. Lamin A/C was detected using a specific primary antibody and linked to an Alexa Fluor 488-conjugated secondary antibody (green). Nucleic acids were stained using DAPI (blue). Evaluation of lamin A/C-positive (**C**) nuclei with nuclear inclusions, (**D**) nuclei with 1–2 or ≥3 nuclear inclusions, (**E**) deformed nuclei, (**F**) nuclear invaginations and (**G**) mild or moderate nuclear invaginations. The quantitative data are presented as mean ± SEM and were obtained by analysing 50 cells per condition from four independent experiments. The statistical analysis was performed using one-way ANOVA followed by Tukey’s multiple comparison test to compare between DM1_1000, DM1_2000 and the control groups. * *p* < 0.05; ** *p* < 0.01; Scale bar = 10 µm; ↑ represents nuclear invaginations; ^ represents nuclear inclusions; DM1—myotonic dystrophy type 1; SEM—standard error of the mean.

**Figure 4:**
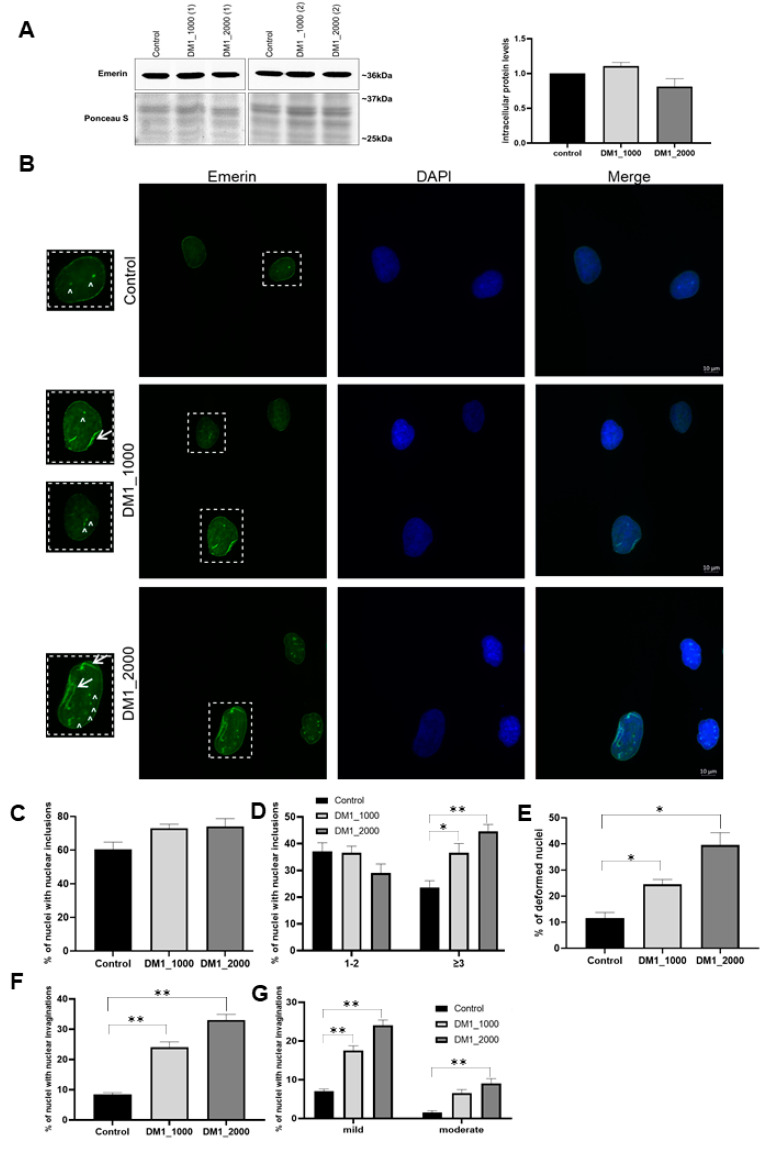
Intracellular protein levels and nuclear localization of emerin in DM1 patient-derived and control fibroblasts. (**A**) Intracellular emerin protein levels in DM1 patient-derived and control fibroblasts. The intracellular protein levels in DM1 patient-derived fibroblasts were estimated in relation to protein levels detected in the control condition and are presented as mean ± SEM of four independent experiments. Ponceau S staining was used to assess gel loading. The statistical analysis was performed using one-way ANOVA followed by Tukey’s multiple comparison test to compare between DM1_1000, DM1_2000 and the control groups. (**B**) Subcellular distribution of emerin in DM1 patient-derived fibroblasts and control was analysed using fluorescence microscopy. Emerin was detected using a specific primary antibody and an anti-mouse Alexa-488-conjugated secondary antibody (green). Nucleic acids were stained using DAPI (blue). Evaluation of emerin-positive (**C**) nuclei with nuclear inclusions, (**D**) nuclei with 1–2 or ≥3 nuclear inclusions, (**E**) deformed nuclei, (**F**) nuclear invaginations and (**G**) mild or moderate nuclear invaginations. The quantitative data are presented as mean ± SEM and were obtained by analysing 50 cells per condition from four independent experiments. The statistical analysis was performed using one-way ANOVA followed by Tukey’s multiple comparison test to compare between DM1_1000, DM1_2000 and the control groups. * *p* < 0.05, ** *p* < 0.01; Scale bar = 10 µm; ↑ represents nuclear invaginations; ^ represents nuclear inclusions. DM1—myotonic dystrophy type 1; SEM—standard error of the mean.

**Figure 5:**
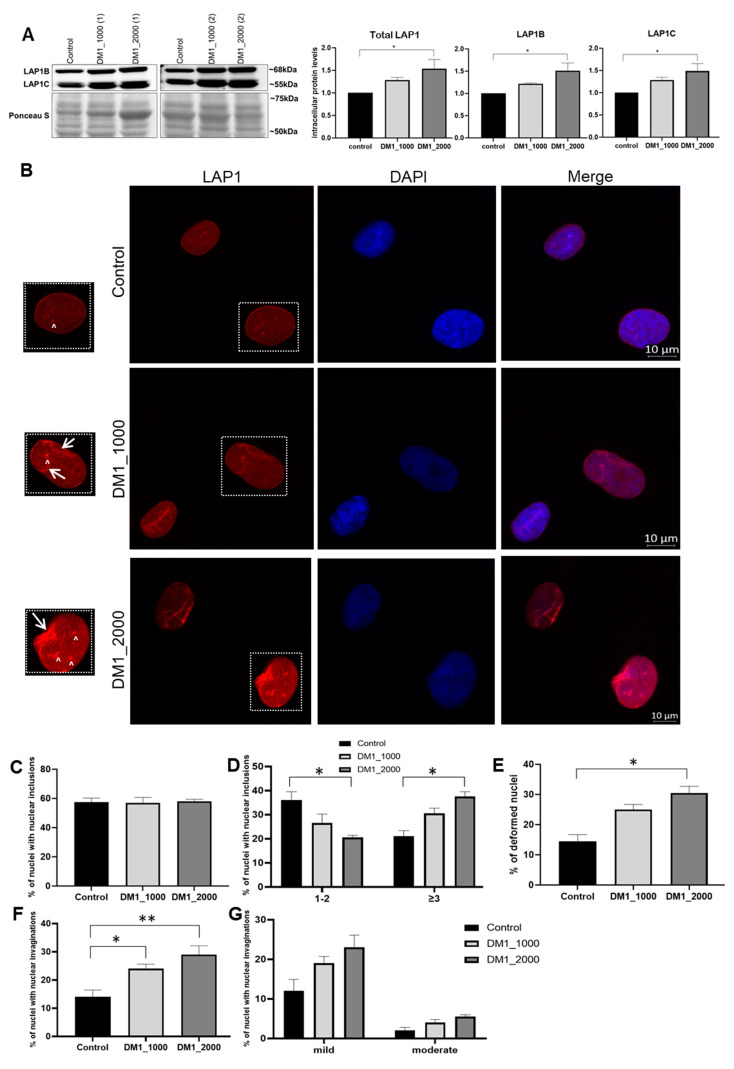
Intracellular protein levels and nuclear localization of LAP1 in DM1 patient-derived and control fibroblasts. (**A**) Total LAP1, LAP1B and LAP1C intracellular protein levels in DM1 patient-derived and control fibroblasts. The intracellular protein levels in DM1 patient-derived fibroblasts were estimated in relation to protein levels detected in the control condition and are presented as mean ± SEM of four independent experiments. Ponceau S staining was used to assess gel loading. The statistical analysis was performed using one-way ANOVA followed by Tukey’s multiple comparison test to compare between DM1_1000, DM1_2000 and the control groups. * *p* < 0.05 (**B**) Subcellular distribution of LAP1 in DM1 patient-derived and control fibroblasts was analysed using fluorescence microscopy. LAP1 was detected using a specific primary antibody linked to an anti-mouse Alexa-594-conjugated secondary antibody (red). Nucleic acids were stained using DAPI (blue). Evaluation of LAP1-positive (**C**) nuclei with nuclear inclusions, (**D**) nuclei with 1–2 or ≥3 nuclear inclusions, (**E**) deformed nuclei, (**F**) nuclear invaginations and (**G**) mild or moderate nuclear invaginations. The quantitative data are presented as mean ± SEM and were obtained by analysing 50 cells per condition from four independent experiments. The statistical analysis was performed using one-way ANOVA followed by Tukey’s multiple comparison test to compare between DM1_1000, DM1_2000 and the control groups. * *p* < 0.05, ** *p* < 0.01; Scale bar = 10 µm; ↑ represents nuclear invaginations; ^ represents nuclear inclusions. DM1—myotonic dystrophy type 1; LAP1—lamin-associated polypeptide 1; SEM—standard error of the mean.

**Figure 6:**
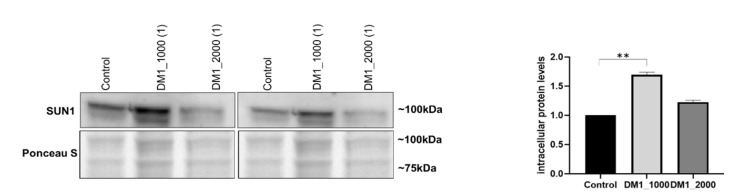
Intracellular protein levels of SUN1 in DM1 patient-derived and control fibroblasts. The intracellular protein levels in DM1 patient-derived fibroblasts were estimated in relation to protein levels detected in the control condition and are presented as mean ± SEM of three independent experiments. Ponceau S staining was used to assess gel loading. The statistical analysis was performed using one-way ANOVA followed by Tukey’s multiple comparison test to compare between DM1_1000, DM1_2000 and the control groups. ** *p* < 0.01; DM1—myotonic dystrophy type 1; SEM—standard error of the mean; SUN—Sad1/Unc-84.

**Figure 7:**
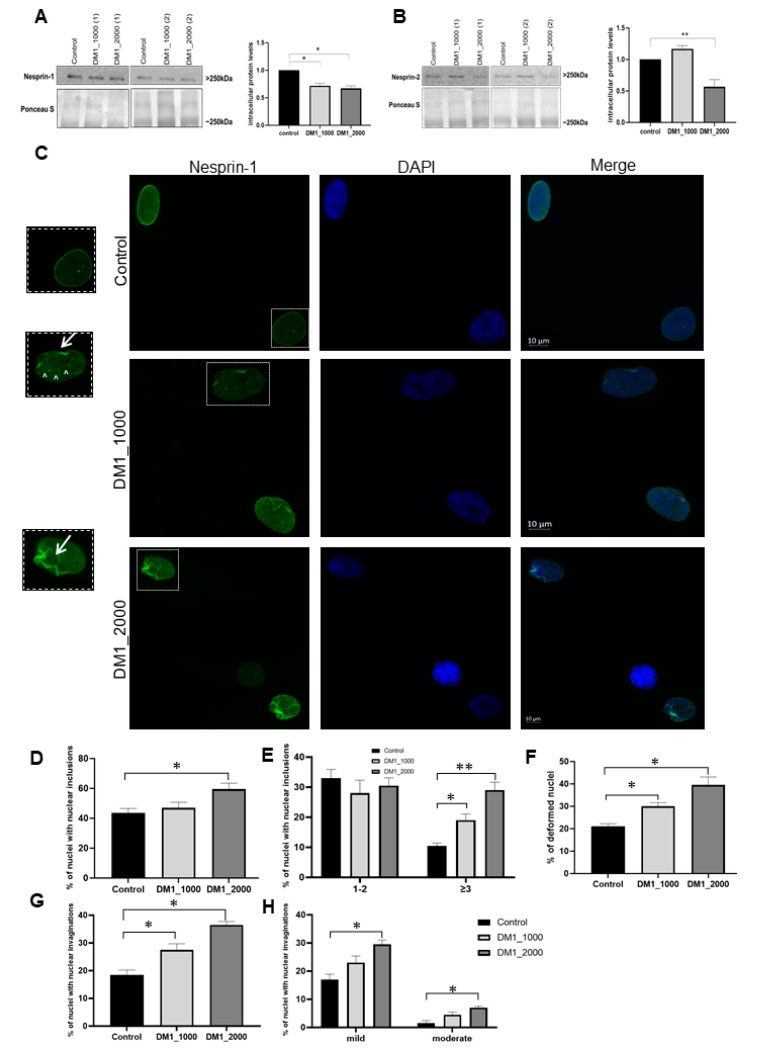
Intracellular protein levels and nuclear localization of nesprin-1 in DM1 patient-derived and control fibroblasts. (**A**) Intracellular nesprin-1 protein levels in DM1 patient-derived and control fibroblasts. The intracellular protein levels in DM1 patient-derived fibroblasts were estimated in relation to protein levels detected in the control condition and are presented as mean ± SEM of four independent experiments. Ponceau S staining was used to assess gel loading. The statistical analysis was performed using one-way ANOVA followed by Tukey’s multiple comparison test to compare between DM1_1000, DM1_2000 and the control groups. * *p* < 0.05. (**B**) Intracellular nesprin-2 protein levels in DM1 patient-derived and control fibroblasts. The intracellular protein levels in DM1 patient-derived fibroblasts were estimated in relation to protein levels detected in the control condition and are presented as mean ± SEM of four independent experiments. Ponceau S staining was used to assess gel loading. The statistical analysis was performed using one-way ANOVA followed by Tukey’s multiple comparison test to compare between DM1_1000, DM1_2000 and the control groups. ** *p* < 0.01 (**C**) Subcellular distribution of nesprin-1 in DM1 patient-derived and control fibroblasts was analysed using fluorescence microscopy. Nesprin-1 was detected using a specific primary antibody and an anti-mouse Alexa-488-conjugated secondary antibody (green). Nucleic acids were stained using DAPI (blue). Evaluation of nesprin-1 positive (**D**) nuclei with nuclear inclusions, (**E**) nuclei with 1–2 or ≥3 nuclear inclusions, (**F**) deformed nuclei, (**G**) nuclear invaginations and (**H**) mild or moderate nuclear invaginations. The quantitative data are presented as mean ± SEM and were obtained by analysing 50 cells per condition from four independent experiments. The statistical analysis was performed using one-way ANOVA followed by Dunnett’s test to compare between DM1_1000, DM1_2000 and the control groups. * *p* < 0.05, ** *p* < 0.01; Scale bar = 10 µm; ↑ represents nuclear invaginations; ^ represents nuclear inclusions. DM1—myotonic dystrophy type 1; SEM—standard error of the mean.

To investigate the intracellular protein levels and subcellular localization of NE proteins in DM1 patient-derived and control fibroblasts, immunoblotting and immunocytochemistry techniques were used, respectively. Essentially, the following NE proteins were evaluated: nuclear lamin protein, namely lamin A/C (Figure 3); three inner nuclear membrane proteins, including emerin (Figure 4), LAP1 (Figure 5) and SUN1 (Figure 6); and two outer nuclear membrane proteins, including nesprin-1 (Figure 7A) and nesprin-2 (Figure 7B).

Regarding the intracellular protein levels of lamin A/C, an increase was observed in the DM1 patient-derived fibroblasts DM1_1000 and DM1_2000 (*p* = 0.0058) in relation to the control fibroblasts, which was more pronounced in fibroblasts with higher CTG repeat length (Figure 3A). The results also demonstrated that lamin A/C was located in the NE and nucleoplasm, and an increase in lamin A/C immunolabelling in DM1 patient-derived fibroblasts was observed (Figure 3B). The increase in the percentage of lamin A/C-positive nuclear inclusions in DM1 patient-derived fibroblasts was evident, with a significant alteration being observed between DM1_2000 and control fibroblasts (*p* = 0.0254) (Figure 3C). The number of DM1 patient-derived fibroblasts’ nuclei with three or more inclusions (≥3) was significantly different between the DM1_2000 and control fibroblasts (*p* = 0.0012) (Figure 3D). 

Concerning deformed nuclei, the DM1 patient-derived fibroblasts showed more lamin A/C-positive deformations than the control fibroblasts, with this increase being significant between the DM1_2000 and control fibroblasts (*p* = 0.0328) (Figure 3E). Regarding nuclear invaginations, DM1_1000 and DM1_2000 fibroblasts had a higher number of nuclear invaginations than the controls, with this difference being significant in the DM1_2000 fibroblasts in comparison to the control (*p* = 0.033) (Figure 3F). The patient-derived fibroblasts (DM1_2000) also demonstrated a significant increase in the number of moderate invaginations compared to the control fibroblasts (*p* = 0.0014) (Figure 3G).

Upon nuclear lamina evaluation, several important alterations were observed in type A lamins, indicating that the NE structure and function could be compromised. Therefore, the subsequent analysis of their functional partners was of paramount importance. The intracellular protein levels of emerin remained apparently unchanged in DM1 patient-derived fibroblasts when compared with control fibroblasts (Figure 4A). Furthermore, our results also demonstrated that emerin was located not only in the NE but also in the nucleoplasm, in which the nuclear inclusions were more evident and in higher number in DM1 patient-derived fibroblasts (Figure 4B–D).

Additionally, the DM1 patient-derived fibroblasts that were immunolabeled for emerin presented a higher percentage of deformed nuclei than the control fibroblasts (DM1_1000 vs. control: *p* = 0.0245; DM1_2000 vs. control: *p* = 0.0032) (Figure 4E). The results also showed that the DM1_1000 (*p* = 0.0040) and DM1_2000 (*p* = 0.0026) fibroblasts presented a significantly higher number of nuclei with invaginations than the controls (Figure 4F). The DM1 patient-derived fibroblasts showed a significant increase in mild (DM1_1000 vs. control: *p* = 0.0033; DM1_2000 vs. control: *p* = 0.0062) and moderate (DM1_2000 vs. control: *p* = 0.0089) invaginations when compared with control-derived fibroblasts (Figure 4G).

LAP1 is another important inner nuclear membrane (INM) protein, belonging to a dynamic and complex network of interactions spanning the perinuclear space and connecting the nuclear lamina, the NE, the cytoskeleton and nucleoskeleton. At least two human LAP1 isoforms are known, namely LAP1B and LAP1C [25,26]. LAP1 interacts with several proteins relevant to this study, such as nuclear lamins and emerin [27,28].

The intracellular protein levels of total LAP1 as well as individual LAP1B and LAP1C isoforms were increased in DM1 patient-derived fibroblasts. Furthermore, this alteration seems to be correlated with CTG repeat length, being statistically significant between DM1_2000 patient-derived fibroblasts and control fibroblasts (total LAP1: *p* = 0.0302; LAP1B: *p* = 0.0137; LAP1C: *p* = 0.0210) (Figure 5A). Our results also showed that LAP1 was not only located in the NE, and an immunostaining of the NE and nucleoplasm was observed in DM1 patient-derived fibroblasts (Figure 5B). However, the number of nuclear inclusions in fibroblasts derived from patients with DM1 were identical (Figure 5C). When analysing the two established categories, it was observed that cells with one and two inclusions tended to decrease in patients with DM1_1000 and DM1_2000 (*p* = 0.0310) when compared to the control fibroblasts. Concomitantly, the presence of three or more nuclear inclusions was significantly increased in DM1_2000 fibroblasts when compared to the control (*p* = 0.0472) (Figure 5D).

The percentage of LAP1-positive deformed nuclei in DM1 patient-derived fibroblasts tended to be higher than in the control fibroblasts, and this increase was more pronounced in fibroblasts with a higher CTG repeat length (DM1_2000 vs. control: *p* = 0.0301) (Figure 5E). However, most deformities observed in LAP1 positive nuclei in patient-derived fibroblasts tended to be mild (Figure 5G).

SUN1 was another protein evaluated from the inner nuclear membrane There was a significant increase in the intracellular protein levels of SUN1 in fibroblasts from patients with DM1_1000 (*p* = 0.0082) compared to the control fibroblasts (Figure 6).

Following the assessment of nuclear lamins and inner nuclear membrane proteins, we carried on with the evaluation of two important outer nuclear membrane proteins, namely nesprin-1 and nesprin-2. SUN1, nesprin-1 and nesprin-2 are the core components of the linker of nucleoskeleton and cytoskeleton (LINC) complex and were therefore evaluated [29].

Regarding nesprin-1, a statistically significant decrease in nesprin-1 intracellular protein levels was observed in DM1_1000 (*p* = 0.0179) and DM1_2000 (*p* = 0.0129) patient-derived fibroblasts in relation to the control fibroblasts (Figure 7A). Concerning nesprin-2, the results showed a significant decrease in the protein intracellular levels in DM1_2000 patient-derived fibroblasts when compared to the control fibroblasts (*p* = 0.0059) (Figure 7B).

Regarding immunocytochemistry, this study demonstrated the NE and nucleoplasm localization of nesprin-1 in DM1 patient-derived fibroblasts (Figure 7C). The results demonstrated an increase in the number of nesprin 1-nuclear inclusions in DM1 patient-derived fibroblasts, being statistically significant between DM1_2000 and control (*p* = 0.0226) (Figure 7D). When we analysed the inclusions by groups, we found that cells with greater than 3 nuclear inclusions tended to increase in DM1 patient-derived fibroblasts when compared to the control-derived fibroblasts (DM1_1000 vs. control, *p* = 0.0281; DM1_2000 vs. control, *p* = 0.0002) (Figure 7E).

Taking into consideration the nuclear deformity, the DM1-derived fibroblast nuclei were significantly more deformed than the control nuclei (DM1_1000 vs. control: *p* = 0.0280; DM1_2000 vs. control: *p* = 0.0287) (Figure 7F). Regarding nesprin-1 positive nuclear invaginations, DM1_1000 (*p* = 0.0280) and DM1_2000 (*p* = 0.0106) patient-derived fibroblasts presented a percentage of nuclei with invaginations significantly superior to the control fibroblasts (Figure 7G). When we distinguished between mild and moderate invaginations, there was an increase in both in patients, which was significant between the control and DM1_2000 for mild (*p* = 0.0498) and moderate invaginations (*p* = 0.044) (Figure 7H).

## 3. Discussion

In this study, we demonstrated that DM1 patient-derived fibroblast nuclei presented with an aberrant nuclear morphology. Further, alterations in NE proteins, namely DMPK, lamin A/C, emerin, LAP1, SUN1, nesprin-1 and nesprin-2, were observed (Figure 1, Figure 2, Figure 3, Figure 4, Figure 5, Figure 6 and Figure 7). Our results showed decreased DMPK intracellular protein levels in DM1 patient-derived fibroblasts (Figure 1). Since the DMPK protein is encoded by the *DMPK* gene, which is abnormally expanded in the 3′UTR region in DM1, these mutant transcripts with abnormal CUG expansions are not efficiently transported to the cytoplasm and accumulate in cell nuclei; therefore, they are not translated into protein [6,10,23,30,31]. Thus, intracellular protein levels of DMPK are reduced in patients with DM1, regardless of the length of the CTG repeat, as shown in Figure 1. This result was particularly important given that alterations in DMPK protein levels in DM1 patient-derived fibroblasts have not been previously reported.

The nuclear architectural alterations, namely nuclear deformation, the number of micronuclei, crossed diameter ratio and nuclear area, were increased in DM1 patient-derived fibroblasts when compared to control fibroblasts, suggesting that these alterations represent relevant features in DM1 (Figure 2). The expanded RNA in DM1 patient-derived fibroblasts may explain the changes in nuclear integrity, since the expanded mutant RNA exerts an action on chromatin dynamics by remodelling [32] and changing the positioning of nucleosomes [33,34,35]. One of the effects of these conformational changes in chromatin is a decrease in autointegration barrier factor (BAF) availability in cells as a consequence of BAF downregulation [36]. In addition, our results regarding DM1 patient-derived fibroblasts revealed a large number of cells with micronuclei (Figure 2C), which may be due to errors in the NE reassembly process after cell division, since an altered reassembly of NE accompanied by the abnormal incorporation of chromosomes may result in the encapsulation of separate and smaller genetic material (i.e., a micronucleus) [37]. Further, BAF deletion has been associated with defects in NE reassembly [37]. This transcription factor interacts with the NE proteins emerin, MAN1, LAP2 and lamin A/C [38,39,40]. Thus, the expanded mutant RNA and BAF interference with these NE proteins may explain the increased growth of deformed nuclei and micronuclei in DM1 patient-derived fibroblasts. Furthermore, it has also been suggested that the linker of nucleoskeleton and cytoskeleton (LINC) complex dampens forces in the NE while preserving nuclear morphology and constraining nuclear expansion, while also being involved in the nuclear positioning and disassembly process of the NE during mitosis [41,42,43,44]. Therefore, alterations in the LINC complex (such as the decrease in intracellular levels of nesprins and the increase in SUN1) may be responsible for the altered localization of nuclei in the cells and the increase in the nuclear area in fibroblasts derived from patients with DM1 [45], which is in accordance with our results regarding nesprins and SUN1protein levels.

In our study, a nuclear lamina protein was also evaluated, namely lamin A/C, which demonstrated increased intracellular levels, localization in the NE and in the nucleoplasm, and nuclear deformations in DM1 patient-derived fibroblasts (Figure 3). This may be correlated with the significantly decreased levels of DMPK protein observed in DM1 patient-derived fibroblasts, since according to a previous study, [46] DMPK seems to be essential in maintaining the stability of NE and strict regulation of DMPK levels is absolutely necessary to stabilize NE structure. Nonetheless, more studies are needed to decipher the role of increased lamin A/C in DM1, and to understand if the increase in lamin A/C intracellular protein levels is an attempt to stabilize the nuclear structure. Regarding the nuclear deformations observed, they may be due to a deregulation of the chromatin organization caused by the abnormal expansion of the CTG repeat. Furthermore, lamin A/C, as well as emerin, are NE proteins that regulate the organization of chromatin, and this regulation is essential for normal cell functioning. However, chromatin can undergo changes in its organization due to DNA damage [47]. When this damage occurs, the cells stop to proliferate and consequently enter into senescence, forming foci of heterochromatin in cell nuclei [48,49]. In the case of DM1, the abnormal expansion of the CTG repeat may have a role in the alteration of the subcellular localization of lamin A/C, and in the higher number of nuclear inclusions and deformations in DM1 patient-derived fibroblasts, leading to chromatin dysregulation and consequently the formation of heterochromatin foci associated with senescence.

The results regarding the three inner nuclear membrane proteins, namely emerin, LAP1 and SUN1, were also interesting. Concerning emerin protein levels, no significant differences between DM1 patient-derived and control fibroblasts were observed (Figure 4A). In addition, we found that emerin in DM1-patients’ nuclei was located at the NE and in the nucleoplasm, accompanied by an increase in the number of inclusions and nuclear deformations (Figure 4B–G). Our results are in agreement with a previous study that also used DM1 fibroblasts as a cell model [20]. The fact that our results did not demonstrate changes in intracellular emerin levels leads us to speculate that the structural changes observed in the nucleus are not correlated with emerin intracellular protein levels, but rather are associated with a destabilized nuclear lamina and other NE proteins. Interestingly, it was previously reported that a destabilized nuclear lamina leads to greater nuclear fragility [50,51], resulting in an increase in deformed nuclei, nuclear breaks accompanied by abnormal chromatin organization and chromatin extrusion [51,52]. LAP1, to our knowledge, has not been previously evaluated in DM1. Our study demonstrated that total LAP1 intracellular levels are increased in DM1 patient-derived fibroblasts, and this increase seems to be correlated with the number of CTG repeat length (Figure 5A). In addition, we observed an incorrect localization of the protein, an increase in nuclear inclusions, and deformed nuclei (Figure 5B–G). LAP1 has been associated with processes regulating the development and maintenance of skeletal muscle and the integrity of the NE [53,54,55]. However, the cause that leads to the increase in this protein was not determined and should be addressed in future studies. Furthermore, LAP1 and torsinA interact with each other, and LAP1 stimulates torsinA activity as an ATPase AAA^+^ [56]. Both proteins have been identified as mediators of the assembly of the LINC complex and are responsible for the localization of nesprins in the NE [57,58,59,60]. Thus, torsinA and LAP1 belong to a dynamic network of interactions that connect the nuclear lamina, the NE and the cytoskeleton [57,58,61]. Thus, the altered localization of LAP1 and the increase in deformed nuclei observed in our study may be associated with an abnormal positioning of the nuclei and/or nuclear deformations resulting from the mechanical stress exerted on cells that have a weakened NE due to alterations in the nuclear lamina and proteins of the LINC complex. In turn, SUN1 is responsible for locating torsinA in the NE [58,60] and is involved in the connection of the nucleoplasm with the cytoskeleton, in nuclear anchorage and in nuclear migration [62,63,64]. Our results demonstrated an increase in SUN1 intracellular levels in DM1 patient-derived fibroblasts when compared to control fibroblasts (Figure 6). Mutations in *SUN1* (with decreased levels of intracellular protein) have been associated with *EMD*, which is histologically manifested by the alteration of nuclear position and, consequently, the degradation of muscle function [65]. Given the alterations in lamin A/C location reported in DM1, SUN1 may be increased due to the impossibility of interacting with lamin A/C, mimicking what occurs in mutated lamin A HGPS fibroblasts and thus sharing mechanisms with other laminopathies. This hypothesis is reinforced by previous results showing that myoblasts from DM1 patients with positive nuclei labeled for SUN1 did not present changes in the localization of this protein [22]. However, it should be noted that these myoblasts and the cells used in this study are distinct models and the results may differ; thus, further studies will be needed to evaluate this hypothesis.

Finally, the two evaluated outer nuclear membranes proteins, namely nesprin-1 and nesprin-2, showed decreased intracellular levels in DM1 patient-derived fibroblasts. This result is in accordance with previous studies using myoblasts and myotubes from patients with DM1, where a tendency for nesprin-1 and -2 to decrease with an increasing number of CTG repeats was reported [22]. We also found that positive nuclei labeled for nesprin-1 showed an altered protein localization, increased number of deformed nuclei and increased number of nuclear inclusions (Figure 7C–H). It was also previously reported that some muscular dystrophies associated with mutations in both *SYNE-1* and *SYNE-2*, the genes encoding nesprin-1 and nesprin-2, respectively, are characterized by abnormal nuclear morphology, micronuclei and fragmented nuclei [15,66], similar to what we observed in our results. These changes are usually due to an incorrect localization of the LINC complex proteins (nesprins and SUN 1/2) or their interactors (lamin A/C) [15,67,68]. Low intracellular levels of nesprins result in a defective interaction of LINC complex proteins and/or complex-associated proteins (for example, emerin, LAP1 and lamin A/C) with nuclear actin. With this function impaired, nuclear positioning, NE architecture, gene expression and maintenance of muscle fibers in patients with muscle diseases are affected [69]. Therefore, the decrease in nesprin-1 intracellular protein levels might be related to the structural changes in the NE (deformed nuclei and nuclear inclusions) observed in our study.

Our results strengthen the hypothesis that NE dysfunction is an important contributor to DM1. Therefore, the identification of the signaling events underlying the NE dysfunction will be of extreme importance for the identification of novel molecular targets for DM1.

## 4. Materials and Methods

### 4.1. Human Samples

Fibroblasts derived from skin biopsies of adult male DM1 donors with different numbers of CTG repeats, and from a healthy control subject, were obtained from the Coriell Institute for Medical Research, Newark, NJ, USA. The clinically affected patients’ cell lines selected for this study included two cell lines with approximately 1000 CTG repeats, referred to as DM1_1000 (1) (GM04033) and DM1_1000 (2) (GM04647), and two cell lines with approximately 2000 CTG repeats, designated DM1_2000 (1) (GM03759) and DM1_2000 (2) (GM03989). The DM1 patient-derived fibroblasts with approximately 1000 and 2000 CTG repeat lengths represented the adult and congenital phenotypes, respectively. In turn, the control cell line used in this study comprised between 5 and 27 CTG repeats (GM02673).

### 4.2. Cell Culture

Fibroblast cultures were maintained in T75 flasks with Dulbecco’s Modified Eagle Medium (DMEM; Gibco, Thermo Fisher Scientific, Waltham, MA, USA) supplemented with 15% fetal bovine serum (FBS; Gibcoᵀᴹ), at 37 °C in a humidified atmosphere with 5% CO_2_. The medium was changed every other day and all washes performed using Dulbecco’s phosphate buffered saline (PBS; Thermo Scientific, Thermo Fisher Scientific, Waltham, MA, USA). Whenever fibroblast cultures reached a confluence of 80–90%, they were subcultured using 0.05% trypsin-EDTA, plated in complete medium and maintained at 37 °C in a CO_2_ incubator [70].

### 4.3. Antibodies

The list of primary and secondary antibodies used for Western blotting and immunocytochemistry is summarized in Table 1.

### 4.4. Immunoblotting

Fibroblast cultures were grown in T75 flasks until they reached a confluence of 80–90%. Cell lysates were collected in 1% sodium dodecyl sulphate (SDS) and boiled at 90 °C for 10 min. The total protein content was quantified using Pierce’s bicinchoninic acid (BCA) protein assay kit (Thermo Scientific, Thermo Fisher Scientific, Waltham, MA, USA). Protein samples were separated on a 5–20% SDS-PAGE gradient gel and electrotransferred onto nitrocellulose membranes. Reversible staining of nitrocellulose membranes with Ponceau S (Sigma-Aldrich, Saint Louis, MO, USA), followed by scanning in a calibrated image densitometer GS-800 (Bio-Rad, San Jose, CA, USA) was performed to assess gel loading [73,74].

For immunoblotting analysis of target proteins, upon blocking in 5% bovine serum albumin (BSA; Nzytech, Lisbon, Portugal)/1× Tris-buffered saline with 0.1% Tween-20 (TBS-T) for 3 h, the membranes were incubated with the primary antibodies (Table 1) in 3% BSA/1× TBS-T for 2 h at room temperature, followed by overnight incubation at 4 °C. On the next day, the membranes were incubated with the appropriate HRP-conjugated secondary antibody (Table 1) in 5% fat-free dry milk/1× TBS-T for 2 h at room temperature. For the detection of target proteins, the enhanced chemioluminescence ECL™ Select Western blotting detection reagent (GE Healthcare, Waukesha, WI, USA) was used, and immunoblots were scanned in a ChemiDoc imaging system (Bio-Rad, Hercules, CA, USA) [27].

The quantification of intracellular protein levels was achieved with ImageLab software (Bio-Rad, Hercules, CA, USA), and Ponceau S staining was used as a protein loading control for data normalization [27]. Relative protein levels were calculated by comparing the DM1 patients’ samples with the control samples.

### 4.5. Immunocytochemistry

Fibroblasts were plated in 6-well plates containing glass coverslips (Corning, New York, NY, USA) at a cell density of 75,000 cells/well for 24 h. Then, cells were fixed using 4% paraformaldehyde for 20 min and permeabilized with 0.2% Triton X-100/1× PBS for 10 min. After blocking with 3% BSA/1× PBS for 1 h, the cells were incubated with specific primary antibodies (Table 1) in 3% BSA/1× PBS for 2 h at room temperature, followed by incubation with the appropriate secondary antibody (Table 1) in 3% BSA/1× PBS for 1 h in the dark. The coverslips were mounted on a microscope slide using Vectashield^®^ mounting medium with 4′,6-diamidino-2-phenolyde (DAPI) (Vector Laboratories, Burlingame, CA, USA) [27,74]. Image acquisition was performed using an epifluorescence microscopy Zeiss AxioImager Z1 (Zeiss, Jena, Germany) motorized microscope equipped with a Plan-ApoCHROMAT 63×/1.4 oil objective lens. Microphotograph images were taken with a digital AxioCam HR3 (soft imaging system).

#### 4.5.1. Morphological Analysis

Two hundred nuclei from each cell line were analyzed. From the morphological point of view, the nuclear form and the number of nuclear inclusions were evaluated. The number of nuclear inclusions was assessed globally and by categories (1–2 inclusions and ≥3 inclusions). Nuclei were considered normal when they presented a typical ring-shaped immunostaining pattern for NE proteins or an ellipsoid shape when stained with DAPI. In turn, nuclei were considered deformed when they presented nuclear alterations/deformations, such as invaginations, blebs, lobes and micronuclei. Additionally, deformed nuclei were subdivided into two different categories according to the presence of mild invaginations (very soft deformations observed) or moderate invaginations (severe deformations observed). Representative images of mild (Figure 3; DM1_2000) and moderate (Figure 5; DM1_2000) invaginations are presented.

#### 4.5.2. Morphometric Analysis

For morphometric analysis, four hundred nuclei from each cell line were evaluated. Quantitative analyses of the circularity ((4π × area)/perimeter^2^), nuclear area and crossed diameter ratio (length/width) were performed automatically using Fiji/ImageJ software.

### 4.6. Statistical Analysis

Statistical analysis was conducted using the GraphPad Prism 9 software (GraphPad Software, San Diego, CA, USA) and data were analyzed using one-way ANOVA followed by Tukey’s multiple comparison test. Quantitative data were presented as mean ± standard error of the mean (SEM) of, at least, three independent experiments. Values of *p* < 0.05 were considered statistically significant.

## 5. Conclusions

In summary, our results clearly demonstrate that nuclear profile and nuclear envelope proteins are altered in DM1-patient derived fibroblasts. Concerning the nuclear profile, increased nuclear area, a high number of deformed nuclei and the high presence of micronuclei were the most prominent alterations observed in DM1 patient-derived fibroblasts.

Regarding the NE protein alterations, the protein levels of lamin A/C, LAP1 and SUN1 were increased, while the levels of emerin and nesprin-1/nesprin-2 remained unaltered and decreased, respectively. Additionally, the results showed an altered localization of these NE proteins, accompanied by the presence of nuclear deformations, including blebs, lobes and/or invaginations that were well correlated with the structural differences in the nuclei observed in DM1-derived fibroblasts.

Our study has strengthened the hypothesis that changes in the NE are important hallmarks of DM1 and supports further studies and the exploitation of NE dysfunction in DM1 as a target for the development of DM1 therapies.

## Figures and Tables

**Table 1:** Primary antibodies used to detect the multiple proteins analyzed by Western blotting and immunocytochemistry.

Antibody	Company	Dilution
Mouse monoclonal anti-lamin A/C (4777T)	Cell Signaling Technology	WB—1:4000 ICC—1:250
Rabbit polyclonal anti-LAP1 (ICC: HPA050546)	Provided by Dr. Dauer [71]	WB—1:20,000
	Atlas Antibodies	ICC—1:150
Mouse monoclonal anti-emerin (sc-25284)	Santa Cruz Biotechnology	WB—1:1000 ICC—1:500
Rabbit polyclonal anti-SUN1	Provided by Ya-Hui Chi [72]	WB—1:2000
Mouse monoclonal anti-nesprin-1 (MANNES1E 8C3)	Developmental Studies Hybridoma Bank	WB—0.4 μg/mL ICC—1.5 μg/mL
Mouse monoclonal anti-nesprin-2 (MANNES2A 11A3)	Developmental Studies Hybridoma Bank	WB—0.3 μg/mL
Mouse monoclonal anti-DMPK (MANDM1 6G8)	Developmental Studies Hybridoma Bank	WB—0.2 ug/mL
Alexa Fluor 488-conjugated goat anti-mouse IgG (A-11001)	Invitrogen	ICC—1:300
Alexa Fluor 594-conjugated goat anti-rabbit IgG (A-11012)	Invitrogen	ICC—1:300
HRP-linked horse anti-mouse IgG (7076)	Cell Signaling Technology	WB—1:10,000
HRP-linked goat anti-rabbit IgG (7074)	Cell Signaling Technology	WB—1:10,000

WB, Western Blotting; ICC, Immunocytochemistry.

## Data Availability

Not applicable.
